# Effectiveness of Internet-Based Telehealth Programs in Patients With Hip or Knee Osteoarthritis: Systematic Review and Meta-Analysis

**DOI:** 10.2196/55576

**Published:** 2024-09-30

**Authors:** Hao-Nan Wang, Pei Luo, Shuyue Liu, Yunyi Liu, Xiao Zhang, Jian Li

**Affiliations:** 1 Sports Medicine Center West China Hospital Sichuan University Chengdu China; 2 Department of Orthopedics and Orthopedic Research Institute West China Hospital Sichuan University Chengdu China; 3 School of Sport Science Beijing Sport University Beijing China; 4 Key Laboratory of Physical Fitness and Exercise Ministry of Education Beijing China; 5 Faculty of Kinesiology University of Calgary Calgary, AB Canada; 6 School of Physical and Occupational Therapy Faculty of Medicine McGill University Montreal, QC Canada

**Keywords:** osteoarthritis, knee, hip, telehealth, telemedicine, telerehabilitation, eHealth, exercise, PRISMA

## Abstract

**Background:**

Osteoarthritis (OA) is a chronic musculoskeletal disease that causes pain, functional disability, and an economic burden. Nonpharmacological treatments are at the core of OA management. However, limited access to these services due to uneven regional local availability has been highlighted. Internet-based telehealth (IBTH) programs, providing digital access to abundant health care resources, offer advantages, such as convenience and cost-effectiveness. These characteristics make them promising strategies for the management of patients with OA.

**Objective:**

This study aimed to evaluate the effectiveness of IBTH programs in the management of patients with hip or knee OA.

**Methods:**

We systematically searched 6 electronic databases to identify trials comparing IBTH programs with conventional interventions for hip and knee OA. Studies were selected based on inclusion and exclusion criteria, focusing on outcomes related to function, pain, and self-efficacy. Standardized mean differences (SMDs) with 95% CIs were calculated to compare outcome measures. Heterogeneity was assessed using *I²* and χ² tests. The methodological quality of the selected studies and the quality of evidence were also evaluated.

**Results:**

A total of 21 studies with low-to-high risk of bias were included in this meta-analysis. The pooled results showed that IBTH has a superior effect on increasing function (SMD 0.30, 95% CI 0.23-0.37, *P*<.001), relieving pain (SMD –0.27, 95% CI –0.34 to –0.19, *P*<.001), and improving self-efficacy for pain (SMD 0.21, 95% CI 0.08-0.34, *P*<.001) compared to the conventional intervention group. Subgroup analysis revealed that IBTH with exercise can significantly alleviate pain and improve function and self-efficacy, but IBTH with cognitive-behavioral therapy only had the effect of reducing pain.

**Conclusions:**

The meta-analysis provides moderate-quality evidence that IBTH programs have a beneficial effect on improving function, relieving pain, and improving self-efficacy compared to conventional interventions in patients with hip or knee OA. Limited evidence suggests that the inclusion of exercise regimens in IBTH programs is recommended.

**Trial Registration:**

PROSPERO CRD42024541111; https://www.crd.york.ac.uk/prospero/display_record.php?RecordID=541111

## Introduction

Osteoarthritis (OA) is a worldwide chronic musculoskeletal disease. It is characterized by pathologic changes in cartilage, bone, synovium, ligament, muscle, and periarticular fat [1]. OA causes chronic pain, joint stiffness, disability, and a subsequent impact on the quality of life [2]. Worldwide, it is estimated that more than 240 million individuals have activity-limiting, symptomatic OA [3]. Among the major joints, the hip and the knee are most commonly affected by OA [4]. An estimated 24% of the general adult population has OA [5]. Both hip OA and knee OA are the leading causes of disability worldwide, with hip OA ranked as the 11th-highest contributor to disability worldwide and knee OA ranked as the 38th highest in term of years lived with disability [6]. OA-related disability results in substantial direct costs and mortality. On average, individuals with knee OA spend approximately US $15,000 in direct medical costs over their lifetimes [7]. There is currently no cure for OA, and to date, most research has primarily focused on treatments that alleviate pain and prevent functional decline. Nonpharmacological strategies, including patient education, cognitive-behavioral therapy (CBT), weight loss, and exercise, constitute the core management for hip and knee OA before resorting to surgical or pharmacological interventions [8-11]. However, the face-to-face delivery of health care services (eg, exercise and CBT) can be prohibitively expensive in terms of time and other costs for patients with incurable OA who require long-term interventions. However, the COVID-19 pandemic has forced us to change our traditional approach to treatment, so we can strike a balance between physical protection and ongoing care for older adults [12,13]. As the prevalence and treatment costs of hip and knee OA increase, there is a growing recognition of the need to identify effective treatment options that provide timely and equitable access to services regardless of location, accessibility, or public health policies, such as lockdown or quarantine, as in the case of the COVID-19 pandemic [14].

Telehealth is a promising health care delivery method that can help improve access to care. According to the World Health Organization (WHO) definition, telehealth refers to the “delivery of health care services, where patients and providers are separated by distance. Telehealth uses Information and Communication Technologies (ICT) for the exchange of information for the diagnosis and treatment of diseases and injuries, research and evaluation, and for the continuing education of health professionals” [15]. Recently, improvements in technology have dramatically increased the accessibility and quality of digitally delivered care. Despite this, telehealth has yet to be widely adopted due to stringent regulatory laws and a lack of supportive payment structures [16]. During the COVID-19 pandemic, social distancing requirements and lockdown restrictions severely impacted face-to-face health services and interventions. Telehealth has the potential to greatly increase patient access to quality, affordable care, while maintaining physical distance for the safety of both patients and providers [17]. For this reason, telehealth has been adopted and accelerated worldwide as a safe and feasible remote health service [18]. From the patient’s perspective, the primary goal of telehealth is to increase access to care and improve the convenience of health care delivery. Telehealth can improve access to health care resources, especially in rural and underdeveloped areas [19]. Telehealth can also significantly reduce the time required to receive medical care, including travel time to health care facilities, time spent in waiting rooms, and time spent actually receiving medical care [20]. Furthermore, telehealth has the potential to contribute to the reduction in health care costs for patients. The implementation of remote monitoring and ongoing management of a patient’s health and chronic conditions can reduce hospitalizations and delay joint replacements for patients with OA [21].

Over the past few decades, researchers have been dedicatedly assessing the effectiveness and feasibility of telehealth interventions, and delivery methods have continued to evolve. In the initial stages of research, texting and calling were used as delivery methods for telehealth interventions. However, the outcomes were not consistently satisfactory. For instance, telephone-based weight loss support with weight management and healthy lifestyle services does not reduce knee pain intensity or weight compared to usual care [22]. The advent of the internet in clinical practice as an information-sharing medium has created many opportunities for innovative interventions for people with chronic conditions and their health care providers [23]. Internet-based telehealth (IBTH) programs provide digital access to health resources and care, facilitating the remote transmission of patient demographic and clinical data and images via online platforms or mobile apps [24]. Telehealth access via the internet provides effective and enriched alternative assessments (eg, gait analysis), structured interventions (eg, exercise), and recreational interventions (eg, rehabilitation games) [25]. In addition, IBTH programs that incorporate interactive and continuous self-monitoring, feedback, and information exchange in self-directed or automated modes can provide educational materials and home-based exercises to replace some or all of the need for physiotherapist instruction [26,27]. However, the role of IBTH for people with OA is challenging as they are mostly older and less likely to have access to the internet and smart devices [23].

To date, emerging evidence suggests that IBTH programs have experienced rapid growth; however, evidence of their effectiveness in hip and knee OA remains unclear. Several reviews and meta-analyses have sought to evaluate the effectiveness of telehealth programs for individuals with OA, but the majority of these studies have focused on the efficacy of telehealth-supported exercise interventions [28,29]. In addition, these studies had limitations because more structured interventions, such as CBT, were not included. A previous meta-analysis showed that internet-based rehabilitation programs may improve pain but not physical function in patients with knee OA [30]. The meta-analysis investigated IBTH programs with multiple structured interventions for knee OA, but the total sample size was too small to allow definite judgments [30]. In addition, further large studies on IBTH for hip and knee OA have been published. Therefore, the purpose of this systematic review and meta-analysis was to evaluate the effectiveness of IBTH programs in function, pain, and self-efficacy in patients with hip or knee OA.

## Methods

### Search Strategy

This systematic review was conducted in accordance with the Preferred Reporting Items for Systematic Reviews and Meta-Analyses (PRISMA) statement, as outlined in Multimedia Appendix 1 [31]. The priori protocol of this study was reviewed and registered in the International Prospective Register of Systematic Reviews (PROSPERO; CRD42024541111) in May 2024. Two review raters (authors HNW and PL) independently performed a systematic literature search to identify relevant studies. The following databases were searched for papers published between January 1, 2000, and May 3, 2024: PubMed, EMBASE, Web of Science, SPORTDiscus (EBSCO), PEDro, and CINAHL. The search string was divided into 3 sections: the first included synonyms for telehealth, the second consisted of synonyms for the knee or the hip joint, and the third pertained to OA. To ensure that at least 1 search term from each section was included in the results, all synonyms within sections were connected with the “OR” operator, and different sections were connected with the “AND” operator. The search strategies for databases are presented in Multimedia Appendix 2.

### Inclusion and Exclusion Criteria

All studies were screened and assessed for eligibility based on the PICOS (Population, Intervention, Comparison, Outcome, and Study) method, in accordance with our specific inclusion and exclusion criteria. Studies were eligible for inclusion if (1) their subjects were diagnosed with OA; (2) they allowed comparisons between telerehabilitation and face-to-face rehabilitation, usual care, waitlisting, and placebo; (3) they contained at least 1 of any objective measures of function, pain, or self-efficacy as the outcome; or (4) they were randomized or quasi-randomized controlled trials (RCTs). Studies were excluded if (1) they were reviews, case reports, conference reports, or observational investigations; (2) participants had undergone a surgical procedure or experienced lower limb trauma; (3) studies or data were duplicated; or (4) studies were published in a language other than English.

### Data Selection and Extraction

Research results were imported into EndNote version 20 (Clarivate Analytics) to remove duplications and facilitate selection. Two review raters (PL and HNW) independently screened the titles and abstracts retrieved using the search strategy. The full texts of all studies considered potentially eligible for inclusion were subsequently retrieved and read independently by the 2 review raters, who then made the final selection. Any discrepancies were resolved through discussion and, when necessary, by the involvement of a third independent rater (author XZ). Data were independently extracted from the included studies by the 2 review raters (HNW and PL), including author, year, patient characteristics, intervention characteristics, duration of treatment, outcomes, and time points, and the extracted variables were revised and checked for accuracy by the 2 review raters. In this study, we analyzed the data at endpoints that measured immediately after the IBTH intervention. In the case of incomplete data from a published paper, we contacted the corresponding author for the raw data of trials.

### Risk of Bias

The PEDro scale was used to evaluate the quality and bias of the studies in this research. [32]. The PEDro scale, which evaluates study eligibility criteria, random allocation, concealed allocation, baseline comparability, blinded subjects, blinded therapists, blinded assessors, adequate follow-up, intention-to-treat (ITT) analysis, between-group comparisons, and point estimates and variability, was considered valid and reliable [33]. Where available, scores were taken directly from the PEDro database [34]. If scores were unavailable, 2 independent reviewers (HNW and PL) assessed the quality of the papers using the PEDro scale. Any disagreements were resolved by discussion with a third reviewer (XZ).

The revised Cochrane Collaboration’s Risk of Bias version 2 (RoB 2) tool for assessing risk of bias in randomized clinical trials was also used to assess the risk of bias in the included studies by 2 independent authors (HNW and PL). The RoB 2 tool contains algorithms that map responses to signaling questions regarding a proposed risk-of-bias judgment for each outcome assessed in a given study [35]. Therefore, assessment criteria were divided into 5 domains: risk of bias from randomization process, bias due to deviations from intended interventions, bias due to missing outcome data, bias in measurement of the outcome, and risk of bias in selection of the reported result. The risk-of-bias judgment for each of the 5 domains was classified as “low risk of bias,” “some concerns,” or “high risk of bias”. The overall risk of bias on a study level was determined according to the classification of the assessment criteria domains, following guidelines from the RoB 2 tool. Any disagreements were resolved by discussion with a third reviewer (XZ).

### Quality of Intervention Reporting

The Template for Intervention Description and Replication (TIDieR) telehealth checklist was used to evaluate the quality of intervention reporting [36]. This checklist was specifically developed for reporting on telehealth interventions evaluated in clinical trials. The checklist consists of 12 items that provide clear and reproducible descriptions of telehealth interventions. The items include a brief name, the rationale (why), materials (what), the procedure (what), the provider (who), the delivery method (how), the location (where), intervention parameters (when and how much), tailoring, modifications, and adherence (how well, both planned and actual):

- Item 1 (brief name): Provide a name or a phrase that describes the intervention.
- Item 2 (why): Describe any rationale, theory, or goal of the elements essential to the intervention.
- Item 3 (what materials): Describe any physical or informational materials used in the intervention, including those provided to participants or used in intervention delivery or in the training of intervention providers. Provide information about where the materials can be accessed.
- Item 4 (what procedures): Describe each of the procedures, activities, or processes used in the intervention, including any enabling or support activities.
- Item 5 (who provided): For each category of intervention provider (eg, psychologist, nursing assistant), describe their expertise, background, and any specific training given.
- Item 6 (how): Describe the modes of delivery (eg, face to face, internet, or telephone) of the intervention and whether it was provided individually or in a group.
- Item 7 (where): Describe the type(s) of location(s) where the intervention occurred, including any necessary infrastructure or relevant features.
- Item 8 (when and how much): Describe the number of times the intervention was delivered and over what period, including the number of sessions; their schedule; and their duration, intensity, or dose.
- Item 9 (tailoring): If the intervention was planned to be personalized, titrated, or adapted, describe what, why, when, and how.
- Item 10 (modifications): If the intervention was modified during the course of the study, describe the changes (what, why, when, and how).
- Item 11 (how well planned): If intervention adherence or fidelity was assessed, describe how and by whom. If any strategies were used to maintain or improve fidelity, describe them.
- Item 12 (how well: actual): If intervention adherence or fidelity was assessed, describe the extent to which the intervention was delivered as planned.

### Data Synthesis

This meta-analysis was conducted using R version 4.2.2 (R Foundation for Statistical Computing) with the *meta* and *Robvis* packages. [37]. The standardized mean difference (SMD) with a 95% CI was used to calculate all continuous data. The effect size was categorized as small (<0.20), moderate (0.21-0.79), or large (>0.80) according to Cohen criteria [38,39]. The Higgins statistic (*I*^2^) was calculated to evaluate heterogeneity. When the number of included studies was 5 or fewer, the fixed-effects model was applied [40]. When the number of included studies was more than 5, the random-effects model was applied if substantial heterogeneity (*I*^2^>50% or *P*<.05) existed; otherwise, the fixed model was used [41]. The significance level was set at *P*<.05. Sensitivity analysis was performed to evaluate the quality and consistency of results by sequentially omitting each study. In addition, publication bias was assessed using funnel plots and Egger tests [42]. The kappa test was used to evaluate the consistency between raters. In addition to the assessment of the overall effect, subgroup analyses were conducted for the type of IBTH intervention, and the interventions were grouped into 3 clusters: (1) exercise, (2) CBT, and (3) mixed. Moreover, subgroup analyses were conducted for the location of OA, and the interventions were grouped into 3 clusters: (1) hip, (2) knee, and (3) hip/knee.

### Quality Assessment

The Grades of Recommendation Assessment, Development, and Evaluation (GRADE) method was used to evaluate the quality of evidence [43]. GRADE assesses the quality of evidence based on the risk of bias, indirectness, inconsistency, imprecision, and the risk of publication bias. The quality of evidence is graded as high, moderate, low, or very low, based on satisfying the following criteria: risk of bias (downgraded if average PEDro scores across studies<7), inconsistency (downgraded if *I*^2^>50 or a single study with N<300), indirectness (downgraded if heterogeneous population or intervention), and imprecision (downgraded if CI>0.25 in either direction or a single study with N<300).

## Results

**Search Results**

**Figure 1:**
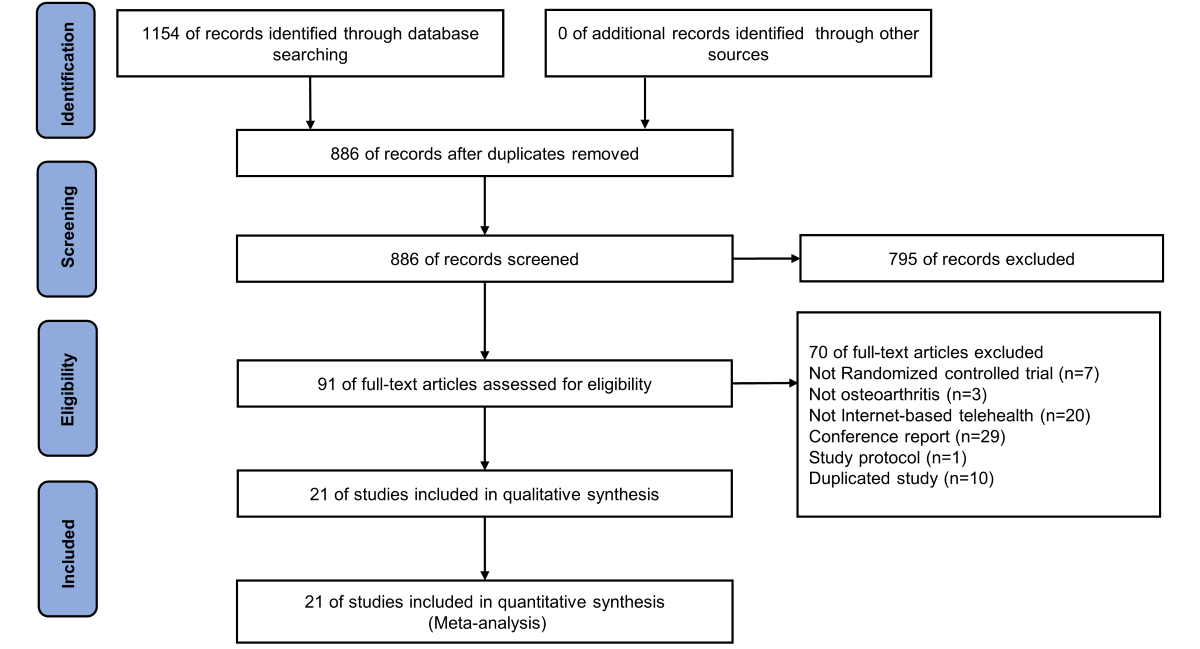
Flow diagram of the literature search and selection following PRISMA. IBTH: internet-based telehealth; OA: osteoarthritis; PRISMA: Preferred Reporting Items for Systematic Reviews and Meta-Analyses; RCT: randomized controlled trial.

The process of paper screening for this study is depicted in Figure 1. A total of 1154 records were identified in the initial search. After removing duplicates, the screening of 886 (76.8%) titles and abstracts retrieved 795 (68.9%) records that did not meet the eligibility criteria. Subsequently, 91 (10.3%) full-text papers were assessed for eligibility. Of these, assessors agreed to include 20 (22%) papers, agreed to exclude 68 (74.7%) papers, and disagreed on whether 3 (3.3%) papers should be included (κ=0.91), indicating good consistency between the 2 raters. Finally, 1 (33.0%) paper was included and 2 (66.7%) were excluded after arbitration by the third rater. This resulted in 21 (44.3%) papers being included in this study.

### Study Characteristics

A total of 21 papers [27,44-63] with 3186 participants were included to evaluate the effectiveness of IBTH in patients with hip or knee OA. The descriptive characteristics of these included studies are summarized in Table 1. All 21 studies were RCTs published between 2013 and 2024. Of these 21 studies, 6 (28.6%) were conducted in Australia, 4 (19%) in the United States, 4 (19%) in the Netherlands, 2 (9.5%) in Turkey, 1 (4.8%) in the United Kingdom, 1 (4.8%) in Brazil, 1 (4.8%) in Greece, 1 (4.8%) in Saudi Arabia, and 1 (4.8%) in Thailand. In addition, 16 (76.2%) trials included patients with knee OA (n=2149, 67.5%), 1 (4.8%) trial included patients with hip OA (n=144, 4.5%), and 4 (19%) trials included patients with hip and knee OA (n=893, 28%). The age of the patients ranged from 55 to 68.5 years. The intervention duration ranged from 6 weeks to 6 months. Furthermore, 18 (85.7%) studies reported short-term (≤3 months) follow-up, while 3 (14.3%) studies reported medium-term (3-6 months) follow-up.

**Table 1:** Summary of the characteristics of studies included in the meta-analysis (N=21).

Author, country	Location of OA^a^ (sample size, n)	Age (years); female/male patients	Comparison	Intervention	Assessment point	Outcomes
Nelligan et al, Australia [27]	Knee (EG^b^: 103; CG^c^: 103)	EG: 60.3; 66/32 CG: 59.0; 60/33	CG: educational information	EG: web-based home exercise plus SMS	6 months	Function: WOMAC^d^ Pain: KOOS^e^ Self-efficacy: ASES^f^
Aily et al, Brazil [44]	Knee (EG: 50; CG:50)	EG: 53; 30/20 CG: 55; 30/20	CG: circuit training	EG: internet-based circuit training	14 weeks	Function: WOMAC Pain: VAS^g^
Alasfour and Almarwani, Saudi Arabia [45]	Knee (EG: 20; CG:20)	EG: 54; 20/0 CG: 55; 20/0	CG: exercise	EG: app-based exercise	6 weeks	Function: WOMAC Pain: VAS
Allen et al, United States [46]	Knee (EG: 142; CG1: 140; CG2: 68)	EG: 65.3; 98/44 CG1:65.7; 100/40 CG2: 64.2; 53/9	CG1: physical therapy CG2: waitlisting	EG: internet-based exercise	4 months	Function: WOMAC Pain: WOMAC
Allen et al, United States [47]	Knee (EG: 230; CG: 115)	EG: 59.9; 36/224 CG: 60.2; 17/98	CG: education	EG: internet-based exercise plus telephone counseling	3 months	Function: WOMAC Pain: WOMAC
Bennell et al, Australia [48]	Hip OA (EG: 73; CG: 71)	EG: 61.2; 45/28 CG: 61.3; 37/34	CG: education	EG: pain-coping skill training	8 weeks	Function: WOMAC Pain: WOMAC Self-efficacy: ASES
Bennell et al, Australia [49]	Knee (EG1: 172; EG2: 175; CG: 67)	EG1: 65.4; 93/79 EG2: 64.1; 89/86 CG:65.3; 45/22	CG: education	EG1: telehealth-delivered exercise EG2: telehealth-delivered diet and exercise	6 months	Function: WOMAC Pain: NRS^h^
Bennell et al, Australia [50]	Knee (EG: 107; CG: 105)	EG: 62.8; 70/37 CG:61.8; 78/27	CG: education	EG: online yoga program	12 weeks	Function: WOMAC Pain: NRS Self-efficacy: ASES
Bossen et al, Netherlands [51]	Hip/knee OA (EG: 100; CG: 99)	EG: 61; 60/40 CG: 63; 69/30	CG: waitlisting	EG: web-based physical activity	3 months	Function: KOOS Pain: KOOS Self-efficacy: ASES
Gohir et al, United Kingdom [52]	Knee (EG: 48; CG: 57)	EG:65.2; 34/14 CG: 68.0; 37/20	CG: usual care	EG: digital exercise program	6 weeks	Function: WOMAC Pain: WOMAC
Hunter et al, Australia [53]	Knee (EG: 112; CG: 105)	EG: 63; 71/41 CG: 66; 60/45	CG: usual care	EG: online exercise and CBT^i^	6 months	Function: KOOS ADL^j^ Pain: NRS
Kloek et al, Netherlands [54]	Hip/knee (EG: 99; CG: 109)	EG: 63.8; 74/35 CG: 62.3; 67/32	CG: usual care	EG: e-exercise	3 months	Function: KOOS/HOOS^k^ ADL Pain: KOOS/HOOS
Moutzouri et al, Greece [55]	Knee (EG: 22; CG: 22)	EG: 65.1; 19/3 CG: 63.5; 17/5	CG: usual care	CG: web-based exercise	6 weeks	Function: KOOS ADL Pain: KOOS
Murphy et al, United States [56]	Knee (EG: 31; CG:15)	EG: 65.1; 19/3 CG: 63.5; 17/5	CG: usual care	CG: online CBT	6 weeks	Function: WOMAC
O’Moore et al, Australia [57]	Knee (EG:49; CG: 20)	EG:63.2; 38/6 CG: 59.68; 17/8	CG: usual care	EG: internet CBT	3 months	Function: WOMAC Pain: WOMAC Self-efficacy: ASES
Pelle et al, Netherlands [58]	Hip/knee (EG: 214; CG: 213)	EG: 62.1; 147/67 CG: 62.1; 159/54	CG: usual care	EG: self-management app with exercise	3 months	Function: KOOS/HOOS ADL Pain: KOOS/HOOS
Rini et al, United States [59]	Knee (EG:58; CG: 55)	EG:68.5; 46/12 CG: 66.7; 45/10	CG: waitlisting	EG: pain-coping skill training	8-10 weeks	Function: AIMS2^l^ Pain: AIMS2 Self-efficacy: ASES
Thiengwittayaporn et al, Thailand [60]	Knee (EG:44; CG: 45)	EG: 62.2; 36/6 CG: 63.0; 37/3	CG: conventional education	EG: mobile app–based self-directed exercise guidance	4 weeks	Function: KOOS ADL Pain: KOOS
Tore et al, Turkey [61]	Knee (EG:24; CG: 24)	EG: 55.9; 21/3 CG: 55.8; 22/2	CG: exercise	EG: exercise via videoconferencing	8 weeks	Function: KOOS ADL Pain: KOOS
Tümtürk et al, Turkey [62]	Knee (EG:29; CG: 28)	EG: 53.6; 24/5 CG: 51.5; 16/12	CG: conventional exercise and education	EG: telerehabilitation-based exercise and education	8 weeks	Function: WOMAC Pain: WOMAC
Weber et al, Netherlands [63]	Hip/knee (EG:29; CG: 28)	EG: 53.6; 24/5 CG: 51.5; 16/12	CG: usual care	EG: app-based exercise, physical activity, and education program	12 weeks	Function: KOOS/HOOS ADL Pain: KOOS/HOOS

^a^OA: osteoarthritis.

^b^EG: experimental group.

^c^CG: control group.

^d^WOMAC: Western Ontario and McMaster Universities Osteoarthritis index.

^e^KOOS: Knee Injury and Osteoarthritis Outcome Score.

^f^ASES: Arthritis Self-Efficacy Scale

^g^VAS: Visual Analogue Scale.

^h^NRS: Numeric Rating Scale.

^i^CBT: cognitive-behavioral therapy.

^j^ADL: activities of daily living.

^k^HOOS: Hip Disability and Osteoarthritis Outcome Score.

^l^AIMS2: Arthritis Impact Measurement Scale 2.

The content of the intervention showed significant variation among the pooled studies. As shown in Table 1, a total of 15 (71.4%) studies used interventions that included an internet-based exercise program [27,46,47,49-55,58,60-63], 4 (19%) studies implemented CBT interventions [48,56,57,59], and 1 (4.8%) study used a combination of an exercise program and a CBT intervention [53].

**Figure 2:**
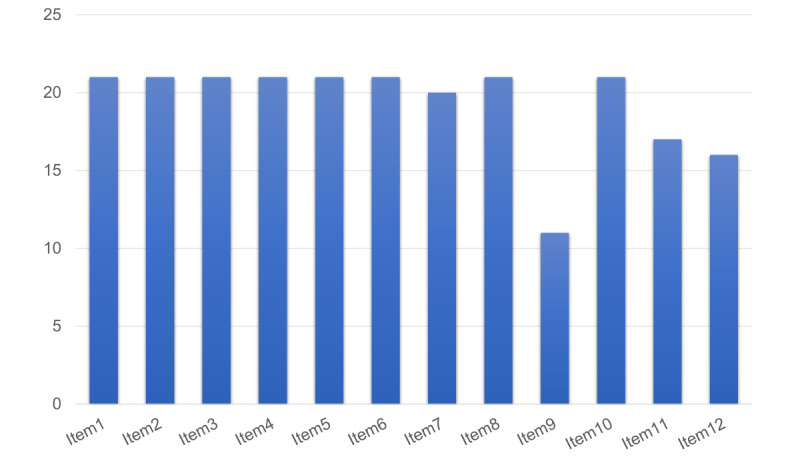
Overview of studies reporting intervention information based on the TIDieR-telehealth checklist. TIDieR: Template for Intervention Description and Replication.

The TIDieR telehealth checklist was assessed for the included studies (Figure 2 and Multimedia Appendix 3). A total of 7(33.3%) studies fulfilled all 12 requirements for the description of a telehealth intervention [46,47,51,52,54,58,60,62]. Only 1 (4.8%) study poorly described the location (item 7) fulfilling the checklist requirements [48]. Around half of the included studies (n=10, 47.6%) [27,44,45,48,50,55-57,59,61] adequately described the tailoring process. All studies reported the actual extent of fidelity for the participants in the intervention. However, 4 (19%) studies [47,53,56,63] inadequately detailed how they planned to maintain or improve fidelity, while 5 (23.8%) studies [47,49,53,56,63] assessed intervention adherence or fidelity. Other items were reported satisfactorily.

### Assessment of Risk of Bias

The risk of bias was assessed for all selected papers on the PEDro scale, and all 21 studies included in the quantitative analysis scored greater than 5, with a maximum score of 10 (Table 2). All included trials used the random allocation method in the study design and comparable outcomes at baseline. A total of 6 (28.6%) trials failed to use allocation concealment [47,54,56,60-62], 2 (9.5%) studies successfully blinded the participants during the trials [48,57], and only 1 (4.8%) study blinded the participants and therapist during the intervention [48], perhaps primarily owing to the characteristics of the interventions. In addition, 11 (52.4%) studies had inadequate blinding of the outcome assessor [27,45,49-52,54,55,58,61,62]. In terms of follow-up, 7 (33.3%) studies did not complete adequate follow-up measurements due to a high attrition rate (>15%) [47,51,52,54,56-58], while 4 (19%) studies did not perform ITT analysis [45,55,56,61]. All studies conducted group comparisons and provided point estimates and variability.

**Figure 3:**
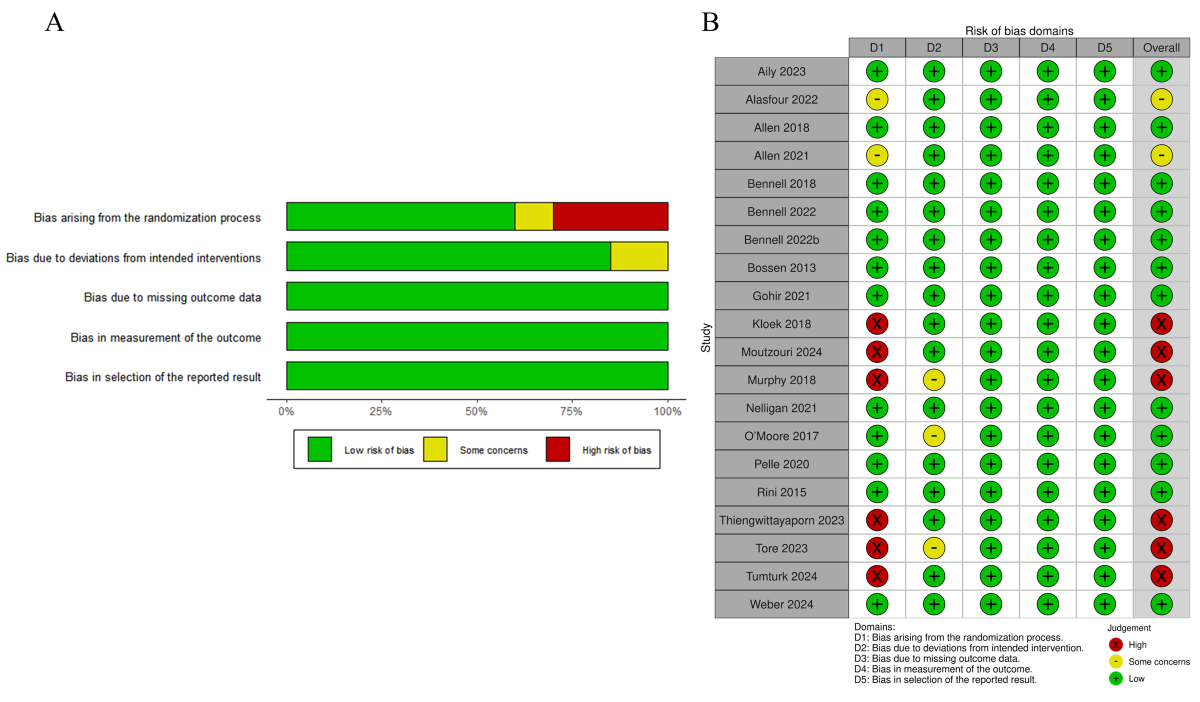
Summary of risk of bias. (A) Risk of bias presented using the Cochrane RoB 2 tool for RCTs and (B) risk of bias as percentages across all included studies. RCT: randomized controlled trial; RoB 2: Risk of Bias version 2.

The methodological quality of the eligible studies was also rated using RoB 2 to assess subjective bias (Figure 3). The overall methodological quality of the studies included in the review was mixed. All studies used methods that we judged to have a low risk of bias to randomly assign participants to either the intervention or the control group. This result was due to the selection criteria for RCTs. Thus, RCTs prevented selection bias and were insured against accidental bias. However, 6 (28.6%) trials did not achieve allocation concealment, which contributed to the high risk of bias in the randomization process [47,54,56,60-62]. A potentially important source of bias in this meta-analysis was the methodology of blinding. Only 1 (4.8%) study could successfully blind the participants and therapists [48]. Around half of the studies (n=10, 47.6%) adopted blinding for the outcome assessor [44,46-48,53,56,57,59,60,63]. Hence, a high risk of measurement of outcomes remained possible for outcomes relying on self-report or objective outcomes by outcome assessors who were not blinded to treatment allocation. The majority of the trials (n=17, 81%) used ITT analysis [27,44,46-54,57-60,62,63], which is an analysis method for solving noncompliance and missing outcomes.

**Table 2:** Methodological classification assessed by the PEDro scale.

Study	Item 2^a^	Item 3^b^	Item 4^c^	Item 5^d^	Item 6^e^	Item 7^f^	Item 8^g^	Item 9^h^	Item 10^i^	Item 11^j^	Total score
Nelligan et al [27]	Yes	Yes	Yes	No	No	No	Yes	Yes	Yes	Yes	7
Aily et al [44]	Yes	Yes	Yes	No	No	Yes	Yes	Yes	Yes	Yes	8
Alasfour and Almarwani [45]	Yes	Yes	No	No	No	No	Yes	No	Yes	Yes	5
Allen et al [46]	Yes	Yes	Yes	No	No	Yes	Yes	Yes	Yes	Yes	8
Allen et al [47]	Yes	No	Yes	No	No	Yes	No	Yes	Yes	Yes	7
Bennell et al [48]	Yes	Yes	Yes	Yes	Yes	Yes	Yes	Yes	Yes	Yes	10
Bennell et al [49]	Yes	Yes	Yes	No	No	No	Yes	Yes	Yes	Yes	7
Bennell et al [50]	Yes	Yes	Yes	No	No	No	Yes	Yes	Yes	Yes	7
Bossen et al [51]	Yes	Yes	Yes	No	No	No	No	Yes	Yes	Yes	6
Gohir et al [52]	Yes	Yes	Yes	No	No	No	No	Yes	Yes	Yes	7
Hunter et al [53]	Yes	Yes	Yes	No	No	Yes	Yes	Yes	Yes	Yes	8
Kloek et al [54]	Yes	No	Yes	No	No	No	No	Yes	Yes	Yes	5
Moutzouri et al [55]	Yes	Yes	Yes	No	No	No	Yes	No	Yes	Yes	6
Murphy et al [56]	Yes	No	Yes	No	No	Yes	No	No	Yes	Yes	5
O’Moore et al [57]	Yes	Yes	Yes	Yes	No	Yes	No	Yes	Yes	Yes	7
Pelle et al [58]	Yes	Yes	Yes	No	No	No	No	Yes	Yes	Yes	6
Rini et al [59]	Yes	Yes	Yes	No	No	Yes	Yes	Yes	Yes	Yes	8
Thiengwittayaporn et al [60]	Yes	No	Yes	No	No	Yes	Yes	Yes	Yes	Yes	7
Tore et al [61]	Yes	No	Yes	No	No	No	Yes	No	Yes	Yes	5
Tümtürk et al [62]	Yes	No	Yes	No	No	No	Yes	Yes	Yes	Yes	6
Weber et al [63]	Yes	Yes	Yes	No	No	Yes	Yes	Yes	Yes	Yes	8

^a^Item 2: Subjects were randomly allocated to groups.

^b^Item 3: Allocation was concealed.

^c^Item 4: Groups were similar at baseline regarding the most important prognostic indicators.

^d^Item 5: There was blinding of all subjects.

^e^Item 6: There was blinding of all therapists who administered the therapy.

^f^Item 7: There was blinding of all assessors who measured at least 1 key outcome.

^g^Item 8: Measures of at least 1 key outcome were obtained from more than 85% of the subjects initially allocated to groups.

^h^Item 9: All subjects for whom outcome measures were available received the treatment or control condition, as allocated, or where this was not the case, data for at least 1 key outcome was analyzed by the intention to treat (ITT).

^i^Item 10: The results of between-group statistical comparisons were reported for at least 1 key outcome.

^j^Item 11: The study provided both point measures and measures of variability for at least 1 key outcome.

### Outcome Measures

#### Primary Outcome

##### Function

**Figure 4:**
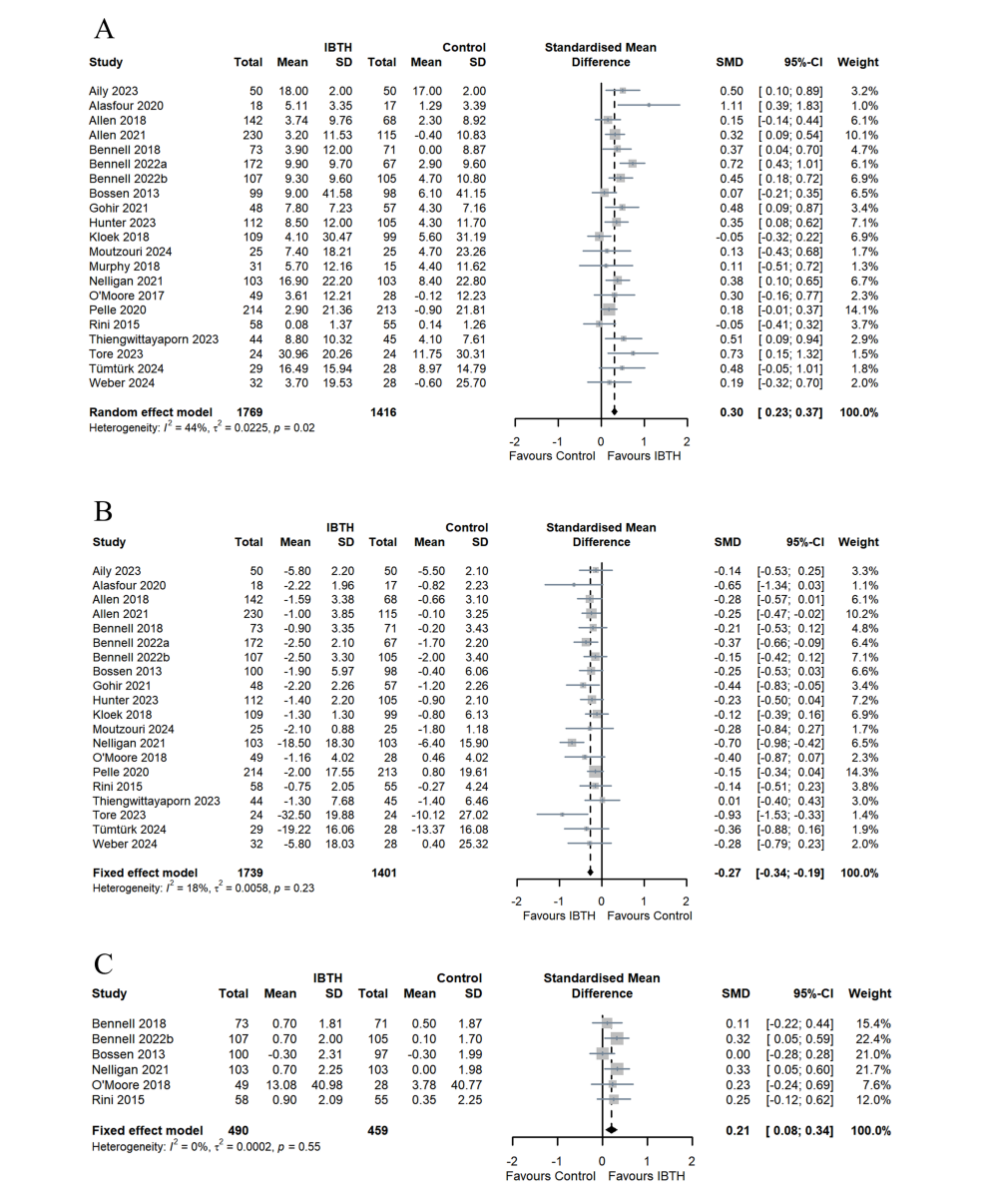
Forrest plots of comparisons of outcomes between IBTH and control groups. (A) Function, (B) pain, and (C) self-efficacy. IBTH: internet-based telehealth; SMD: standardized mean difference.

All 21 studies [27,44-63] evaluated the effects of IBTH compared to control groups. Significant heterogeneity was detected among the included trials (*I*^2^=44%, *P*=.02). Therefore, a random-effects model was used. Participants with hip or knee OA in the IBTH groups had higher improvement in function (SMD 0.30, 95% CI 0.23-0.37, *P*<.001), as shown in Figure 4. The GRADE scale established that the certainty of evidence for function comparisons was low (Multimedia Appendix 4). There was no significant subgroup difference in improving function based on the intervention and the period of follow-up. There was no significant difference between IBTH with CBT and the control group concerning improvement of function (SMD 0.02, 95% CI –0.01 to 0.40, *I*^2^=0). In addition, IBTH was not effective in improving function in studies on patients with mixed hip and knee OA (Multimedia Appendix 5).

#### Secondary Outcomes

##### Pain

A total of 20 (95.2%) studies [27,44-55,57-63] provided data on the pain intensity for IBTH compared to control groups. There was substantial heterogeneity in the pooled studies (*I*^2^=18%, *P*=.23). Hence, a fixed-effects model was adopted. Participants with hip or knee OA in the IBTH groups had a better effect of pain relief than those in the control groups (SMD –0.27, 95% CI –0.34 to –0.19, *P*<.001), as shown in Figure 4. The certainty of GRADE evidence for pain was low (Multimedia Appendix 4). Subgroup analyses indicated there was no significant subgroup difference in pain relief based on the intervention, location, and period of follow-up (Multimedia Appendix 5).

##### Self-Efficacy

Of the 21 studies, 6 (28.6%) [27,48,50,51,57,59] reported outcomes of self-efficacy in both IBTH and control groups. A fixed-effects model was applied in the meta-analysis due to low heterogeneity (*I*^2^=0, *P*=.55). The results indicated that the IBTH groups showed significant improvement in self-efficacy (SMD 0.21, 95% CI 0.08-0.34, *P*<.001) compared to the control groups (Figure 4). The certainty of GRADE evidence for self-efficacy was assessed as high (Multimedia Appendix 4). There was no significant subgroup difference in increasing self-efficacy based on the intervention and the period of follow-up. However, IBTH with CBT significantly improved the self-efficacy of patients with hip or knee OA (SMD 0.18, 95% CI –0.03 to 0.40, *I*^2^=0; Multimedia Appendix 5).

### Sensitivity Analysis

Sensitivity analysis, involving the sequential omission of individual studies, was conducted to evaluate the impact of each study on the overall outcomes. The removal of any specific study did not alter the significance or substantial heterogeneity of the aggregated results pertaining to function, pain, and self-efficacy. Consequently, the sensitivity analysis affirmed that the findings related to significance and heterogeneity were robust.

### Publication Bias

Funnel plots and Egger tests were performed to evaluate publication bias, as illustrated in Multimedia Appendix 6. The funnel plots of the outcomes indicated a potential publication bias in small trials, while Egger tests found that such bias was negligible (*P*>.05) in this meta-analysis.

## Discussion

### Principal Findings

In this meta-analysis, we included 21 studies to evaluate the effects of IBTH programs on patients with hip or knee OA. Low-quality evidence was found to suggest that IBTH programs are more effective in relieving pain and improving function compared to the control group. High-quality evidence showed that IBTH programs have greater efficacy in promoting self-efficacy than control interventions. Subgroup analyses indicated that IBTH (either exercise or CBT) is beneficial in reducing pain intensity compared to control interventions. However, IBTH with CBT may improve function and self-efficacy. The role of telehealth in knee OA therapy was consistent with the results of the primary analysis, but for hip or knee/hip OA, results were inconsistent. The results of this systematic review and meta-analysis support the effectiveness of IBTH programs in improving clinical outcomes in patients with hip or knee OA.

The effectiveness of telehealth programs in lower extremity OA has been the subject of investigation in previous studies. Wang et al [64] investigated the effectiveness of telehealth interventions compared with usual care or no intervention in patients with OA who had undergone total knee replacement (TKR) or total hip replacement (THR). They found that telehealth interventions could improve pain and functional mobility in patients who underwent TKR but had no effect on patients undergoing THR. Two other systematic reviews with meta-analysis addressed a similar question, focusing on the effectiveness of telehealth-based exercise [29,65]. The findings showed that telehealth-based exercise is beneficial in reducing pain and improving function in knee OA. Yang et al [65] found that a 3-month telehealth-based exercise intervention using web and smartphone apps was associated with better pain relief and physical function. This finding is consistent with our subgroup analysis for IBTH with exercise for patients with hip or knee OA. Xie et al [30] investigated the effectiveness of internet-based rehabilitation programs specifically in patients with knee OA. The authors concluded that internet-based rehabilitation programs are effective in reducing pain but not improving the function of patients with knee OA. These differences may be explained by differences in inclusion criteria. The most important differences in eligibility criteria were that we included populations with both hip OA and knee OA, while Xie et al [30] focused only on the latter. We also strictly excluded studies published in conference abstracts because of the absence of full text and complete data, whereas they did not. In addition, only 1 study of IBTH for hip OA was included in this study, so more studies on hip OA are needed in the future for more robust results.

Subgroup analyses were performed to assess the impact of disparate intervention methods in IBTH. The results of our subgroup analyses demonstrated that IBTH with exercise can alleviate pain and improve function and self-efficacy. The effects of exercise on symptoms and function have been well studied in hip and knee OA [66,67]. IBTH with exercise may help with OA symptoms due to the reduced excitability of the motor cortex after exercise, which leads to a decrease in motor evoked potentials, thus reducing pain [68]. IBTH integrated with exercise contributes to an increase in muscle strength, a decrease in extension impairments, and an improvement in proprioception, leading to an improvement in the function of patients with OA [69]. In addition, improved self-efficacy results from exercise can also relieve pain and function. Self-efficacy refers to the belief in one’s ability to successfully perform a behavior to achieve a desired outcome [70]. A previous study has shown that patients with higher levels of arthritis self-efficacy tend to perceive pain stimuli as less unpleasant and have greater pain tolerance [71]. Somers et al [72] found that patients with OA who possess higher arthritis self-efficacy may be better able to tolerate pain. This increased tolerance may increase their likelihood of engaging in weight management behaviors, such as exercise, that are beneficial for improving function. Our findings show emerging evidence that IBTH with exercise results in greater pain reduction, which is consistent with previous studies. In addition to exercise, our subgroup analyses also included the intervention method of CBT. In this systematic review, the included studies on IBTH with CBT were intended to help patients with OA manage pain by self-regulating thoughts, feelings, and behaviors [73]. These interventions delivered through face-to-face methods are effective in managing OA pain [11,74]. Internet-delivered CBT is effective in multiple pain management outcomes, including disability and depression [57,75]. However, we found that IBTH with CBT is ineffective in improving function. One possible explanation is that functional impairment is related to joint degeneration and injury. CBT is a psychological intervention beneficial for pain management, but it has little direct effect on improving physical function. Therefore, IBTH with exercise may be more effective in improving function, and the incorporation of exercise protocols into IBTH programs is recommended.

Our study highlights the potential nonpharmacological treatment strategies that can be delivered via the IBTH method. Nonpharmacological treatment strategies, which serve as the core treatment, are among the most common treatments for patients with OA. Compared with conventional face-to-face delivery methods, treatment delivered via IBTH offers advantages such as lower time consumption, convenience, and lower cost [76], potentially improving adherence to the intervention. These strategies include internet automated/self-paced therapeutic exercise programs, web-based programs to promote daily physical activity, internet-based pain-coping skill training, and internet-based CBT programs. In particular, exercise programs in IBTH allow for individualization and progression [46]. Gohir et al [52] established the progression of exercises during the intervention, adjusting them according to complexity, load, and difficulty in relation to each participant’s response after completing the exercise.

Although telehealth for patients with OA is now well established, future studies can still focus on areas such as remote assessment, remote monitoring, and the integration of novel technologies into IBTH programs. Remote physiotherapy assessment of musculoskeletal disorders via IBTH has been proven feasible and reliable [77], including assessments of the knee [78] and the hip complex [79]. However, remote assessment is more appropriate for follow-up sessions rather than initial consultations, as physical examinations, blood sampling, and radiographic imaging may be required to make a definitive diagnosis in some cases [80]. Monitoring a patient’s health status and interventions is key to a supervised telehealth system. Self-managed telehealth can be facilitated by monitoring health conditions with wearable devices, including measurements such as continuous blood pressure, electrocardiogram, oxygen saturation, and body temperature [81]. For internet-delivered exercise, older patients can use posture and motion monitoring technologies, such as depth cameras and wearable systems, to improve the quality of movement, ensure the effectiveness of exercise, and prevent injury [82-84]. As technology has evolved, the benefits of virtual reality (VR) have been demonstrated across a wide range of conditions [85], making VR attractive to patients with musculoskeletal conditions [86]. In VR interventions, patients interact with the virtual environment to simulate real-life activities, and the rehabilitation process can be gamified [87]. Telehealth via VR can increase patient motivation and improve treatment adherence [88]. As the majority of patients with OA are older adults, the usability of VR-based telehealth should be ensured. As more straightforward platforms have been developed, patients only need a computer or a smartphone to connect to VR via the internet [89]. In addition, some telehealth programs are beginning to use automated programs for dynamic intervention. If telehealth can be combined to enable remote assessment, monitoring, and dynamic adjustments based on patient status, it will provide continuous 24/7 monitoring and intervention, leading to significant cost savings [90].

An improvement in the quality of intervention reporting is warranted. Many of the studies included in this review described intervention details poorly, particularly with regard to the location and intervention parameters (eg, frequency and duration) used in the interventions. These deficiencies may make it infeasible for clinicians to reliably replicate or implement the interventions in future research or practice. For telehealth-based exercise, the American College of Sports Medicine (ACSM) recommends prescribing an exercise program with a variety of parameters [91], including frequency, intensity, time, type, volume, pattern, and progression (FITT-VPP). In addition, the Consensus on Exercise Reporting Template (CERT) has been developed to provide guidance for reporting exercise programs [92]. For IBTH with CBT, specific treatment sessions, frequency, and adherence should be reported, especially for unguided treatment [75]. Hence, researchers should consider using these tools, in addition to the TIDieR telehealth checklist [36,93], when designing and reporting telehealth interventions in the future.

### Strength and Limitations

This systematic review selected papers from a wide range of electronic databases, increasing the comprehensiveness of our findings. This study extends previous systematic reviews in comparable populations. Our review included more eligible studies, which allowed us to perform subgroup analyses to determine the influence of telehealth interventions and locations of OA. Therefore, this meta-analysis may provide valuable insights for evidence-based practice among clinicians and therapists.

We performed procedural reviews and an extensive database search. However, it is possible that the search missed some papers due to omission of the gray literature and selective reporting bias, which is a potential limitation. The analyses of different interventions in IBTH programs (eg, exercise, behavior therapy, or mixed) were not performed due to the limited number of included studies. Thus, more high-quality RCTs with larger sample sizes are needed, especially for hip OA. In addition, the outcome measures that were pooled and synthesized were subjective measures. Our ability to pool data from objective measures was limited in this review due to the heterogeneous outcomes identified from the included studies. None of the studies included a long-term intervention, making it impossible to evaluate the effects of different durations of IBTH interventions on patients with OA. Finally, the results were limited to pooled homogeneous outcome measures due to the variety of different measures used to assess pain and function in the included studies.

### Conclusion

This meta-analysis provided low-to-high quality evidence that IBTH programs have a beneficial effect on improving function, pain, and self-efficacy compared to conventional interventions in patients with hip or knee OA. Limited evidence supports that IBTH with exercise may significantly alleviate pain and improve function and self-efficacy. Therefore, it is recommended that exercise protocols be incorporated into IBTH programs. More high-quality, large-scale evidence is needed to further investigate the therapeutic effects of IBTH.

## Appendix

Multimedia Appendix 1 PRISMA checklist. PRISMA: Preferred Reporting Items for Systematic Reviews and Meta-Analyses.

Multimedia Appendix 2 Search strategy for the systematic review.

Multimedia Appendix 3 TIDieR-telehealth checklist for reporting of interventions in the included studies. TIDieR-telehealth: Template for Intervention Description and Replication.

Multimedia Appendix 4 Summary of findings on the efficacy and safety of IBTH compared to control group using the GRADE scale. GRADE: Grades of Recommendation Assessment, Development, and Evaluation; IBTH: internet-based telehealth.

Multimedia Appendix 5 Summary of subgroup meta-analyses.

Multimedia Appendix 6 Funnel plot and *P* value of Egger tests of the IBTH group versus the control group for (A) function, (B) pain, and (C) self-efficacy. IBTH: internet-based telehealth.

## Abbreviations

ADL: activities of daily living
AIMS2: Arthritis Impact Measurement Scale 2
ASES: Arthritis Self-Efficacy Scale
CBT: cognitive-behavioral therapy
GRADE: Grades of Recommendation Assessment, Development, and Evaluation
HOOS: Hip Disability and Osteoarthritis Outcome Score
IBTH: internet-based telehealth
ITT: intention to treat
KOOS: Knee Injury and Osteoarthritis Outcome Score
NRS: Numeric Rating Scale
OA: osteoarthritis
PRISMA: Preferred Reporting Items for Systematic Reviews and Meta-Analyses
RCT: randomized controlled trial
RoB 2: Risk of Bias version 2
SMD: standardized mean difference
THR: total hip replacement
TIDieR: Template for Intervention Description and Replication
TKR: total knee replacement
VAS: Visual Analogue Scale
VR: virtual reality
WOMAC: Western Ontario and McMaster Universities Osteoarthritis index
