# Supplementary material for: Effectiveness of Internet-Based Telehealth Programs in Patients With Hip or Knee Osteoarthritis: Systematic Review and Meta-Analysis
Source: J Med Internet Res. 2024 Sep 30;26:e55576. doi: 10.2196/55576 (PMC11474128; doi:10.2196/55576)
Supplement: Multimedia Appendix 2 [file jmir_v26i1e55576_app2.docx]

**Multimedia Appendix 2**

Search strategy for systematic review

Database: Pubmed <2000 to 2024>

Date searched: May 3, 2024

Search Strategy:

--------------------------------------------------------------------------------

#1 ("Telemedicine"[MeSH Terms]) OR ("Telemedicine"[Title/Abstract]) OR ("Telehealth"[Title/Abstract]) OR ("eHealth"[Title/Abstract]) OR ("mHealth"[Title/Abstract]) OR ("Telerehabilitation"[MeSH Terms]) OR ("Telerehabilitation"[Title/Abstract]) OR ("Remote Rehabilitation"[Title/Abstract]) OR ("Telenursing"[MeSH Terms]) OR ("Telenursing"[Title/Abstract]) OR ("Videoconferencing"[MeSH Terms]) OR ("Videoconferencing"[Title/Abstract]) OR ("Exercise"[MeSH Terms]) OR ("Exercise"[Title/Abstract]) OR ("Cognitive Behavioral Therapy"[MeSH Terms]) OR ("Cognitive Behavioral Therapy"[Title/Abstract]) (592709)

#2 ("Internet"[MeSH Terms]) OR ("Internet"[ Title/Abstract]) OR ("Internet-Based Intervention"[MeSH Terms]) OR ("Internet-Based Intervention"[Title/Abstract]) OR ("Web-based Intervention"[Title/Abstract]) OR ("Online Intervention"[Title/Abstract]) OR ("Mobile Applications"[MeSH Terms]) OR ("Mobile Applications"[Title/Abstract]) OR ("Mobile App"[Title/Abstract]) OR ("Smartphone App"[Title/Abstract]) OR ("App"[Title/Abstract]) OR ("Mobile"[Title/Abstract]) (319125)

#3 (("Osteoarthritis"[MeSH Terms]) OR ("Osteoarthritis"[Title/Abstract]) OR ("Degenerative Arthritis"[Title/Abstract)) AND (("Hip"[MeSH Terms]) OR ("Hip"[Title/Abstract]) OR ("Hip joint"[MeSH Terms]) OR ("Hip joint"[Title/Abstract]) OR ("Knee"[MeSH Terms]) OR ("Knee"[Title/Abstract]) OR ("Knee joint"[MeSH Terms]) OR ("Knee joint"[Title/Abstract])) OR ("Osteoarthritis, Hip"[MeSH Terms]) OR ("Osteoarthritis, Hip "[Title/Abstract]) OR ("Hip Osteoarthritis"[Title/Abstract]) OR ("Osteoarthritis Of Hip"[Title/Abstract]) OR ("Osteoarthritis, Hip"[MeSH Terms]) OR ("Knee Osteoarthritis"[Title/Abstract]) OR ("Osteoarthritis Of Knee"[Title/Abstract]) OR ("Osteoarthritis, Knee "[Title/Abstract]) (64985)

#4 #1 AND #2 AND #3 (198)

Database: Embase <2000 to 2024 >

Date searched: 3 May 2024

Search Strategy:

1. exp telehealth/ or exp telecare/ or exp telemedicine/ or exp telenursing/ or exp teletherapy/ or exp telerehabilitation/ or exp exercise/ or exp psychotherapy/ (830524)

2. (telehealth or ehealth or e-health or tele-health or e-care or e-healthcare or tele-care or telecare or telemedicine or teletherapy or tele-therapy or telerehabilitation or e-rehabilitation or tele-rehabilitation or telenursing or tele-nursing or videoconference$ or mhealth or m-health or mobile health or exercise or psychotherapy).ti,ab. (551001)

3. exp Internet/ or exp web-based intervention/ (133865)

4. (Internet or web-based intervention or internet-based intervention or internet-intervention or online-based intervention or online-intervention or web intervention or online-intervention or web or mobile or online).ti,ab. (771195)

5. exp hip osteoarthritis/ (14873)

6. exp knee osteoarthritis/ (46759)

7. (osteoarthritis or osteoarthrosis).ti,ab. (129932)

8. (hip or knee).ti,ab. (415658)

9. 7 and 8 (70539)

10. 1 or 2 (1009359)

11. 3 or 4 (813452)

12. 5 or 6 or 9 (87036)

13. 10 and 11 and 12 (592)

14. limit 13 to yr="2000 - 2024" (591)

Database: Web of Science <2000 to 2024>

Date searched: May 3, 2024

Search Strategy:

--------------------------------------------------------------------------------

#1 TS = ("Telemedicine" OR "Telehealth" OR "eHealth" OR "mHealth" OR "Telerehabilitation" OR "Remote Rehabilitation" OR "Telenursing" OR "Videoconferencing" OR "Exercise" OR "Cognitive Behavioral Therapy") (622066)

#2 TS = ("Internet" OR "Internet-Based Intervention" OR "Web-based Intervention" OR "Online Intervention") (426777)

#3 TS = ((("Osteoarthritis" OR "Degenerative Arthritis") AND ("Hip" OR "Hip joint" OR "Knee" OR "Knee joint")) OR "Hip Osteoarthritis" OR "Osteoarthritis Of Hip" OR "Osteoarthritis, Hip" OR "Knee Osteoarthritis" OR "Osteoarthritis Of Knee" OR "Osteoarthritis, Knee") (73607)

#4 #1 AND #2 AND #3 (88)

Database: SPORTDiscus (EBSCO) <2000 to 2024>

Date searched: May 2, 2024

Search Strategy:

--------------------------------------------------------------------------------

S1 (SU "Telehealth") OR (SU "Telemedicine") OR (SU "Telenursing ") OR (SU "Exercise") OR (SU "Psychotherapy ") (145059)

S2 TI (Telemedicine OR Telehealth OR eHealth OR mHealth OR Telerehabilitation OR Remote Rehabilitation OR Telenursing OR Videoconferencing OR Exercise OR Psychotherapy) OR AB (Telemedicine OR Telehealth OR eHealth OR mHealth OR Telerehabilitation OR Remote Rehabilitation OR Telenursing OR Videoconferencing OR Exercise OR Psychotherapy) (152911)

S3 (SU "Internet") OR (SU "Internet-Based Intervention") (7410)

S4 TI (Internet OR web-based intervention OR internet-based intervention OR internet-intervention OR online-based intervention OR online-intervention OR web intervention OR online-intervention OR web OR mobile OR online) OR AB (Internet OR web-based intervention OR internet-based intervention OR internet-intervention OR online-based intervention OR online-intervention OR web intervention OR online-intervention OR web OR mobile OR online) (44114)

S5 (SU "osteoarthritis, hip") OR (SU "osteoarthritis, knee") (8997)

S6 TI (Hip Osteoarthritis OR Osteoarthritis Of Hip OR Osteoarthritis, Hip OR Knee Osteoarthritis OR Osteoarthritis Of Knee OR Osteoarthritis, Knee) OR AB (Hip Osteoarthritis OR Osteoarthritis Of Hip OR Osteoarthritis, Hip OR Knee Osteoarthritis OR Osteoarthritis Of Knee OR Osteoarthritis, Knee) (6127)

S7 S1 OR S2 (216154)

S8 S3 OR S4 (46049)

S9 S5 OR S6 (6299)

S10 S7 AND S8 AND S9 (46)

Database: PEDro <2000 to 2024>

Date searched: May 3, 2024

Search Strategy:

--------------------------------------------------------------------------------

Abstract & Title: (Telehealth) OR (Telemedicine) OR (Telerehabilitation)

AND

Problem: pain

AND

Subdiscipline: musculoskeletal

AND

Method: clinical trial

AND

Published Since: 2000

(171)

Database: CINAHL (EBSCO) <2000 to 2024>

Date searched: May 2, 2024

Search Strategy:

--------------------------------------------------------------------------------

S1 (MH "Telemedicine") OR (MH "Telerehabilitation") OR (MH "Telenursing") OR (MH "Exercise") OR (MH "Psychotherapy") (34815)

S2 TI (Telemedicine OR Telehealth OR eHealth OR mHealth OR Telerehabilitation OR Remote Rehabilitation OR Telenursing OR Videoconferencing OR Exercise OR Psychotherapy) OR AB (Telemedicine OR Telehealth OR eHealth OR mHealth OR Telerehabilitation OR Remote Rehabilitation OR Telenursing OR Videoconferencing OR Exercise OR Psychotherapy) (50588)

S3 (MH "Internet") OR (MH "Internet-Based Intervention") (20545)

S4 TI (Internet OR web-based intervention OR internet-based intervention OR internet-intervention OR online-based intervention OR online-intervention OR web intervention OR online-intervention OR web OR mobile OR online) OR AB (Internet OR web-based intervention OR internet-based intervention OR internet-intervention OR online-based intervention OR online-intervention OR web intervention OR online-intervention OR web OR mobile OR online) (77474)

S5 (MH "osteoarthritis, hip") OR (MH "osteoarthritis, knee") (3408)

S6 TI (Hip Osteoarthritis OR Osteoarthritis Of Hip OR Osteoarthritis, Hip OR Knee Osteoarthritis OR Osteoarthritis Of Knee OR Osteoarthritis, Knee) OR AB (Hip Osteoarthritis OR Osteoarthritis Of Hip OR Osteoarthritis, Hip OR Knee Osteoarthritis OR Osteoarthritis Of Knee OR Osteoarthritis, Knee) (2510)

S7 S1 OR S2 (71576)

S8 S3 OR S4 (86512)

S9 S5 OR S6 (4022)

S10 S7 AND S8 AND S9 (60)
