# Supplementary material for: Effectiveness of Internet-Based Telehealth Programs in Patients With Hip or Knee Osteoarthritis: Systematic Review and Meta-Analysis
Source: J Med Internet Res. 2024 Sep 30;26:e55576. doi: 10.2196/55576 (PMC11474128; doi:10.2196/55576)
Supplement: Multimedia Appendix 3 [file jmir_v26i1e55576_app3.docx]

**Multimedia Appendix 3**

TIDieR-telehealth checklist for reporting of intervention in included studies

| TIDieR-telehealth  Item | 1  Brief name | 2  Why | 3  What  (materials) | 4  What  (procedures) | 5  Who  provided | 6  How | 7  Where | 8  When and  how much | 9  Tailoring | 10  Modifications | 11  How well  (planned) | 12  How well  (actual) |
| --- | --- | --- | --- | --- | --- | --- | --- | --- | --- | --- | --- | --- |
| Aily et al [1] | ✓^a^ | ✓ | ✓ | ✓ | ✓ | ✓ | ✓ | ✓ | ✗^b^ | ✓ | ✓ | ✓ |
| Alasfour and Almarwani [2] | ✓ | ✓ | ✓ | ✓ | ✓ | ✓ | ✓ | ✓ | ✗ | ✓ | ✓ | ✓ |
| Allen et al [3] | ✓ | ✓ | ✓ | ✓ | ✓ | ✓ | ✓ | ✓ | ✓ | ✓ | ✓ | ✓ |
| Allen et al [4] | ✓ | ✓ | ✓ | ✓ | ✓ | ✓ | ✓ | ✓ | ✓ | ✓ | ✗ | ✗ |
| Bennell et al [5] | ✓ | ✓ | ✓ | ✓ | ✓ | ✓ | ✗ | ✓ | ✗ | ✓ | ✓ | ✓ |
| Bennell et al [6] | ✓ | ✓ | ✓ | ✓ | ✓ | ✓ | ✓ | ✓ | ✓ | ✓ | ✓ | ✗ |
| Bennell et al [7] | ✓ | ✓ | ✓ | ✓ | ✓ | ✓ | ✓ | ✓ | ✗ | ✓ | ✓ | ✓ |
| Bossen et al [8] | ✓ | ✓ | ✓ | ✓ | ✓ | ✓ | ✓ | ✓ | ✓ | ✓ | ✓ | ✓ |
| Gohir et al [9] | ✓ | ✓ | ✓ | ✓ | ✓ | ✓ | ✓ | ✓ | ✓ | ✓ | ✓ | ✓ |
| Hunter et al [10] | ✓ | ✓ | ✓ | ✓ | ✓ | ✓ | ✓ | ✓ | ✓ | ✓ | ✗ | ✗ |
| Kloek et al [11] | ✓ | ✓ | ✓ | ✓ | ✓ | ✓ | ✓ | ✓ | ✓ | ✓ | ✓ | ✓ |
| Moutzouri et al [12] | ✓ | ✓ | ✓ | ✓ | ✓ | ✓ | ✓ | ✓ | ✗ | ✓ | ✓ | ✓ |
| Murphy et al [13] | ✓ | ✓ | ✓ | ✓ | ✓ | ✓ | ✓ | ✓ | ✗ | ✓ | ✗ | ✗ |
| Nelligan et al [14] | ✓ | ✓ | ✓ | ✓ | ✓ | ✓ | ✓ | ✓ | ✗ | ✓ | ✓ | ✓ |
| O'Moore et al [15] | ✓ | ✓ | ✓ | ✓ | ✓ | ✓ | ✓ | ✓ | ✗ | ✓ | ✓ | ✓ |
| Pelle et al [16] | ✓ | ✓ | ✓ | ✓ | ✓ | ✓ | ✓ | ✓ | ✓ | ✓ | ✓ | ✓ |
| Rini et al [17] | ✓ | ✓ | ✓ | ✓ | ✓ | ✓ | ✓ | ✓ | ✗ | ✓ | ✓ | ✓ |
| Thiengwittayaporn et al [18] | ✓ | ✓ | ✓ | ✓ | ✓ | ✓ | ✓ | ✓ | ✓ | ✓ | ✓ | ✓ |
| Tore et al [19] | ✓ | ✓ | ✓ | ✓ | ✓ | ✓ | ✓ | ✓ | ✗ | ✓ | ✓ | ✓ |
| Tümtürk et al [20] | ✓ | ✓ | ✓ | ✓ | ✓ | ✓ | ✓ | ✓ | ✓ | ✓ | ✓ | ✓ |
| Weber et al [21] | ✓ | ✓ | ✓ | ✓ | ✓ | ✓ | ✓ | ✓ | ✓ | ✓ | ✗ | ✗ |

^a^✓, item sufficiently described in the study;

^b^✗, inadequately or not described.

## References

1. Aily JB, de Noronha M, Approbato Selistre LF, Ferrari RJ, White DK, Mattiello SM. Face-to-face and telerehabilitation delivery of circuit training have similar benefits and acceptability in patients with knee osteoarthritis: a randomised trial. *J Physiother*. 2023 Oct; 69(4):232-239. PMID: 37684147. doi: 10.1016/j.jphys.2023.08.014.
2. Alasfour M, Almarwani M. The effect of innovative smartphone application on adherence to a home-based exercise programs for female older adults with knee osteoarthritis in Saudi Arabia: a randomized controlled trial. *Disabil Rehabil*. 2022 Jun; 44(11):2420-2427. PMID: 33103499. doi: 10.1080/09638288.2020.1836268.
3. Allen KD, Arbeeva L, Callahan LF, Golightly YM, Goode AP, Heiderscheit BC, et al. Physical therapy vs internet-based exercise training for patients with knee osteoarthritis: results of a randomized controlled trial. *Osteoarthr Cartil*. 2018 Mar; 26(3):383-396. PMID: 29307722. doi: 10.1016/j.joca.2017.12.008.
4. Allen KD, Woolson S, Hoenig HM, Bongiorni D, Byrd J, Caves K, et al. Stepped exercise program for patients with knee osteoarthritis : a randomized controlled trial. *Ann Intern Med*. 2021; 174(3):298-307. PMID: CN-02228999. doi: 10.7326/M20-4447.
5. Bennell KL, Nelligan RK, Rini C, Keefe FJ, Kasza J, French S, et al. Effects of internet-based pain coping skills training before home exercise for individuals with hip osteoarthritis (HOPE trial): a randomised controlled trial. *Pain*. 2018 Sep; 159(9):1833-1842. PMID: 29794609. doi: 10.1097/j.pain.0000000000001281.
6. Bennell KL, Lawford BJ, Keating C, Brown C, Kasza J, Mackenzie D, et al. Comparing video-based, telehealth-delivered exercise and weight loss programs with online education on outcomes of knee osteoarthritis : a randomized trial. *Ann Intern Med*. 2022 Feb; 175(2):198-209. PMID: 34843383. doi: 10.7326/m21-2388.
7. Bennell KL, Schwartz S, Teo PL, Hawkins S, Mackenzie D, McManus F, et al. Effectiveness of an unsupervised online yoga program on pain and function in people with knee osteoarthritis : a randomized clinical trial. *Ann Intern Med*. 2022 Oct; 175(10):1345-1355. PMID: 36122378. doi: 10.7326/m22-1761.
8. Bossen D, Veenhof C, Van Beek KE, Spreeuwenberg PM, Dekker J, De Bakker DH. Effectiveness of a web-based physical activity intervention in patients with knee and/or hip osteoarthritis: randomized controlled trial. *J Med Internet Res*. 2013 Nov 22; 15(11):e257. PMID: 24269911. doi: 10.2196/jmir.2662.
9. Gohir SA, Eek F, Kelly A, Abhishek A, Valdes AM. Effectiveness of internet-based exercises aimed at treating knee osteoarthritis: the iBEAT-OA randomized clinical trial. *JAMA Netw Open*. 2021 Feb 1; 4(2):e210012. PMID: 33620447. doi: 10.1001/jamanetworkopen.2021.0012.
10. Hunter DJ, Bowden JL, Hinman RS, Egerton T, Briggs AM, Bunker SJ, et al. Effectiveness of a new service delivery model for management of knee osteoarthritis in primary care: a cluster randomized controlled trial. *Arthr Care Res (Hoboken)*. 2023 Jun; 75(6):1320-1332. PMID: 36205225. doi: 10.1002/acr.25037.
11. Kloek CJJ, Bossen D, Spreeuwenberg PM, Dekker J, de Bakker DH, Veenhof C. Effectiveness of a blended physical therapist intervention in people with hip osteoarthritis, knee osteoarthritis, or both: a cluster-randomized controlled trial. *Phys Ther*. 2018 Jul 1; 98(7):560-570. PMID: 29788253. doi: 10.1093/ptj/pzy045.
12. Moutzouri M, Koumantakis GA, Hurley M, Kladouchou AG, Gioftsos G. Effectiveness of a web-guided self-managed telerehabilitation program enhanced with outdoor physical activity on physical function, physical activity levels and pain in patients with knee osteoarthritis: a randomized controlled trial. *J Clin Med*. 2024 Feb 6; 13(4). PMID: 38398248. doi: 10.3390/jcm13040934.
13. Murphy SL, Janevic MR, Lee P, Williams DA. Occupational therapist-delivered cognitive-behavioral therapy for knee osteoarthritis: a randomized pilot study. *Am J Occup Ther*. 2018 Sep/Oct; 72(5):7205205040p1-p9. PMID: 30157016. doi: 10.5014/ajot.2018.027870.
14. Nelligan RK, Hinman RS, Kasza J, Crofts SJC, Bennell KL. Effects of a self-directed web-based strengthening exercise and physical activity program supported by automated text messages for people with knee osteoarthritis: a randomized clinical trial. *JAMA Intern Med*. 2021 Jun 1; 181(6):776-785. PMID: 33843948. doi: 10.1001/jamainternmed.2021.0991.
15. O'Moore K A, Newby JM, Andrews G, Hunter DJ, Bennell K, Smith J, et al. Internet cognitive-behavioral therapy for depression in older adults with knee osteoarthritis: a randomized controlled trial. *Arthr Care Res (Hoboken)*. 2018 Jan; 70(1):61-70. PMID: 28426917. doi: 10.1002/acr.23257.
16. Pelle T, Bevers K, van der Palen J, van den Hoogen FHJ, van den Ende CHM. Effect of the dr. Bart application on healthcare use and clinical outcomes in people with osteoarthritis of the knee and/or hip in the Netherlands; a randomized controlled trial. *Osteoarthr Cartil*. 2020 Apr; 28(4):418-427. PMID: 32119972. doi: 10.1016/j.joca.2020.02.831.
17. Rini C, Porter LS, Somers TJ, McKee DC, DeVellis RF, Smith M, et al. Automated internet-based pain coping skills training to manage osteoarthritis pain: a randomized controlled trial. *Pain*. 2015; 156(5):837-848. PMID: CN-01152749. doi: 10.1097/j.pain.0000000000000121.
18. Thiengwittayaporn S, Wattanapreechanon P, Sakon P, Peethong A, Ratisoontorn N, Charoenphandhu N, et al. Development of a mobile application to improve exercise accuracy and quality of life in knee osteoarthritis patients: a randomized controlled trial. *Arch Orthop Trauma Surg*. 2023 Feb; 143(2):729-738. PMID: 34453570. doi: 10.1007/s00402-021-04149-8.
19. Tore NG, Oskay D, Haznedaroglu S. The quality of physiotherapy and rehabilitation program and the effect of telerehabilitation on patients with knee osteoarthritis. *Clin Rheumatol*. 2023 Mar; 42(3):903-915. PMID: 36279075. doi: 10.1007/s10067-022-06417-3.
20. Tümtürk İ, Bakırhan S, Özden F, Gültaç E, Kılınç CY. Effect of telerehabilitation-based exercise and education on pain, function, strength, proprioception, and psychosocial parameters in patients with knee osteoarthritis: a randomized controlled clinical trial. *Am J Phys Med Rehabil*. 2024 Mar 1; 103(3):222-232. PMID: 37678215. doi: 10.1097/phm.0000000000002335.
21. Weber F, Kloek C, Stuhrmann S, Blum Y, Grüneberg C, Veenhof C. Usability and preliminary effectiveness of an app-based physical activity and education program for people with hip or knee osteoarthritis - a pilot randomized controlled trial. *Arthr Res Ther*. 2024 Apr 10; 26(1):83. PMID: 38600607. doi: 10.1186/s13075-024-03291-z.
