# Supplementary material for: Effectiveness of Internet-Based Telehealth Programs in Patients With Hip or Knee Osteoarthritis: Systematic Review and Meta-Analysis
Source: J Med Internet Res. 2024 Sep 30;26:e55576. doi: 10.2196/55576 (PMC11474128; doi:10.2196/55576)
Supplement: Multimedia Appendix 4 [file jmir_v26i1e55576_app4.docx]

**Multimedia Appendix 4**

| Outcomes | Effects of interventions (95% CI) | | Number of  participants (studies) | Certainty of the evidence  (GRADE) | Comment |
| --- | --- | --- | --- | --- | --- |
|  | IBTH group | Control group |  |  |  |
| Function | The mean function in the intervention group was 0.32 higher (CI: 0.23 to 0.39) | / | 3185/21 | ⊕⊕  Low^a,b^ | / |
| Pain | The mean pain in the intervention group was 0.29 lower (CI: -0.36 to -0.21) | / | 3140/20 | ⊕⊕  Low^a,b^ | / |
| Self-efficacy | The mean pain in the intervention group was 0.21 higher (CI: 0.08 to 0.34) | / | 949/6 | ⊕⊕⊕⊕  High | / |

Summary of findings for the efficacy and safety of IBTH compared to control group (GRADE).

SMD, standardized mean difference; RR, Risk Ratio; CI, confidence intervals; IBTH, Internet-based Telehealth; GRADE: The Grades of Recommendation Assessment, Development, and Evaluation

a Limitations in study design (average PEDro scores across studies as< 7).

b Inconsistency of results (substantial heterogeneity: *I*^2^>50% or P < .05).

c Imprecision (lower confidence interval spanned an effect size of 0.5).
