# Supplementary material for: Effectiveness of Internet-Based Telehealth Programs in Patients With Hip or Knee Osteoarthritis: Systematic Review and Meta-Analysis
Source: J Med Internet Res. 2024 Sep 30;26:e55576. doi: 10.2196/55576 (PMC11474128; doi:10.2196/55576)
Supplement: Multimedia Appendix 5 [file jmir_v26i1e55576_app5.docx]

**Multimedia Appendix 5**

Summary of subgroup meta-analyses

| **Outcomes** | **Number of studies** | **Effect size** | **ES (95%CI)** | **Heterogeneity(*I*^2^)** | **Chi-square (χ^2^)** |
| --- | --- | --- | --- | --- | --- |
| **Pain** |  |  |  |  |  |
| Intervention |  |  |  |  | 0.24 |
| Exercise | 16 | -0.27 | -0.35 to -0.20 | 32% |  |
| CBT | 3 | -0.23 | -0.44 to -0.01 | 0% |  |
| Mixed treatment | 1 | -0.23 | -0.50 to 0.04 | N/A |  |
| Location |  |  |  |  | 3.17 |
| Knee | 15 | -0.31 | -0.40 to -0.22 | 27% |  |
| Hip | 1 | -0.21 | -0.53 to -0.12 | N/A |  |
| Hip/Knee | 4 | -0.17 | -0.30 to -0.04 | 0% |  |
| **Function** |  |  |  |  |  |
| Intervention |  |  |  |  | 1.16 |
| Exercise | 16 | 0.31 | 0.23 to 0.39 | 53% |  |
| CBT | 4 | 0.20 | -0.01 to 0.40 | 0% |  |
| Mixed treatment | 1 | 0.35 | 0.08 to 0.62 | N/A |  |
| Location |  |  |  |  | 12.51 |
| Knee | 16 | 0.38 | 0.30 to 0.47 | 30% |  |
| Hip | 1 | 0.37 | 0.04 to 0.70 | N/A |  |
| Hip/Knee | 4 | 0.10 | -0.30 to 0.23 | 0% |  |
| **Self-efficacy** |  |  |  |  |  |
| Intervention |  |  |  |  | 0.07 |
| Exercise | 3 | 0.22 | 0.06 to 0.38 | 43% |  |
| CBT | 3 | 0.18 | -0.03 to 0.40 | 0% |  |
| Location |  |  |  |  | 3.74 |
| Knee | 3 | 0.30 | 0.14 to 0.46 | 0% |  |
| Hip | 1 | 0.11 | -0.22 to 0.44 | N/A |  |
| Hip/Knee | 1 | 0.00 | -0.28 to 0.28 | N/A |  |

CBT, cognitive-behavioral therapy
