# Supplementary material for: Effectiveness of Internet-Based Telehealth Programs in Patients With Hip or Knee Osteoarthritis: Systematic Review and Meta-Analysis
Source: J Med Internet Res. 2024 Sep 30;26:e55576. doi: 10.2196/55576 (PMC11474128; doi:10.2196/55576)
Supplement: Multimedia Appendix 6 [file jmir_v26i1e55576_app6.docx]

**Multimedia Appendix 6**


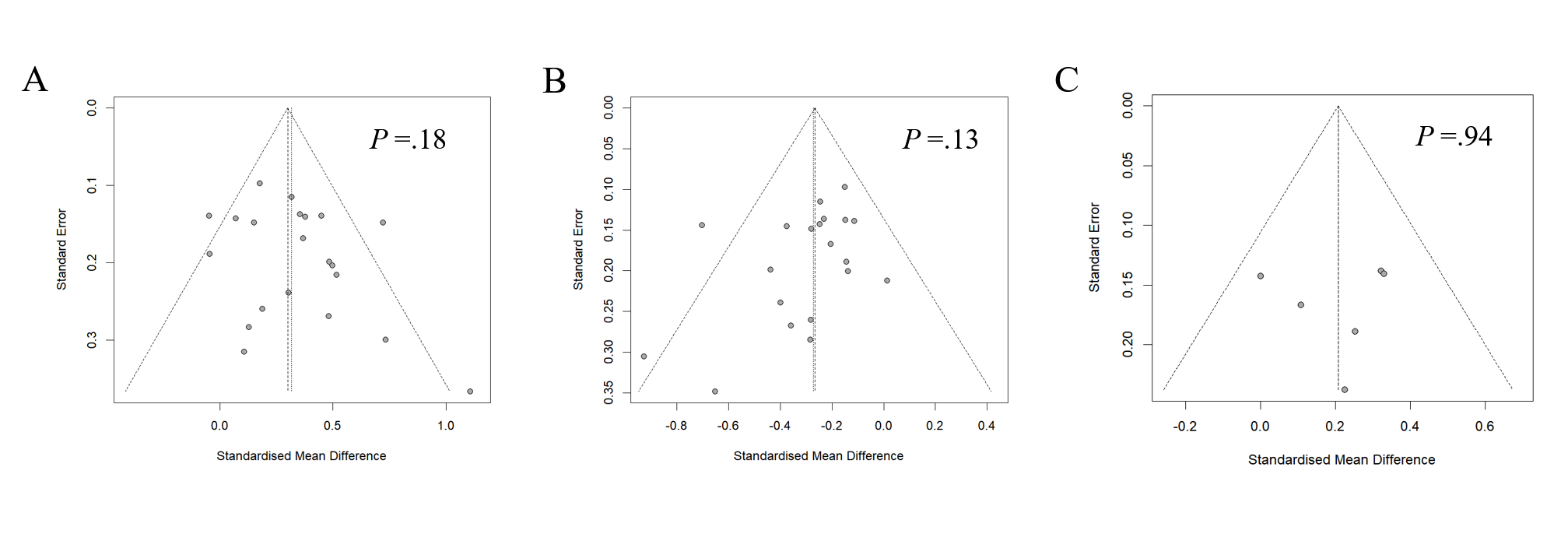


Funnel plot and P value of Egger’s test of IBTH group versus control group for (A) function; (B) pain; (C) self-efficacy.
